# Increased telomere length and proliferative potential in peripheral blood mononuclear cells of adults of different ages stimulated with concanavalin A

**DOI:** 10.1186/1471-2318-13-99

**Published:** 2013-09-24

**Authors:** Blanca Murillo-Ortiz, Froylán Albarrán-Tamayo, Sergio López-Briones, Sandra Martínez-Garza, Luis Benítez-Bribiesca, Diego Arenas-Aranda

**Affiliations:** 1 Unidad de Investigación en Epidemiología Clínica, Unidad Médica de Alta Especialidad (UMAE) No. 1 Bajío, Instituto Mexicano del Seguro Social (IMSS), León, Guanajuato, Mexico; 2 Departamento de Investigaciones Médicas, Universidad de Guanajuato, León, Guanajuato, Mexico; 3 Unidad de Investigación Médica en Enfermedades Oncológicas, CMN, SXXI, IMSS, México, DF, Mexico; 4 Unidad de Investigación en Genética Humana, Hospital de Pediatría, Centro Médico Nacional Siglo XXI, IMSS, México, DF, Mexico

**Keywords:** Telomere length, Proliferative potential, Peripheral blood mononuclear cells

## Abstract

**Background:**

Recently, a direct correlation with telomere length, proliferative potential and telomerase activity has been found in the process of aging in peripheral blood cells. The objective of the study was to evaluate telomere length and proliferative potential in peripheral blood mononuclear cells (PBMCs) after stimulation with Concanavalin A (ConA) of young adults compared with older adults.

**Methods:**

Blood samples were obtained from 20 healthy young males (20–25 years old) (group Y) and 20 males (60–65 years old) (group O). We compared PBMC proliferation before and after stimulation with ConA. DNA was isolated from cells separated before and after culture with ConA for telomeric measurement by real-time polymerase chain reaction.

**Results:**

*In vitro* stimulation of PBMCs from young subjects induced an increase of telomere length as well as a higher replicative capacity of cell proliferation. Samples from older adults showed higher loss of telomeric DNA (*p* = 0.03) and higher levels of senescent (≤6.2 kb) telomeric DNA (*p* = 0.02) and displayed a marked decrease of proliferation capacity. Viability cell counts and CFSE tracking in 72-h-old cell cultures indicated that group O PBMCs (CD8+ and CD4+ T cells) underwent fewer mitotic cycles and had shorter telomeres than group Y (*p* = 0.04).

**Conclusions:**

Our findings confirm that telomere length in older-age adults is shorter than in younger subjects. After stimulation with ConA, cells are not restored to the previous telomere length and undergo replicative senescence. This is in sharp contrast to the response observed in young adults after ConA stimulation where cells increase in telomere length and replicative capacity. The mechanisms involved in this phenomenon are not yet clear and merit further investigation.

## Background

Aging is associated with deterioration of physical and mental functions as well as increased morbidity and mortality. Its mechanism is not fully understood but a number of factors are involved such as hormonal imbalances, oxidative stress, metabolic changes, etc. At the cellular level, evidence suggest that aging is associated with lower immune reactivity and decreased numbers of circulating CD4+ T and B lymphocyte subsets [1]. Recently, a direct correlation with telomere length and telomerase activity has been found in the process of aging.

Telomeres are structures located at the extreme ends of chromosomes and are considered indicators of biological age. Early studies showed the essential role of telomeres in the protection of chromosome integrity [2]. These nucleoprotein caps are maintained by the enzyme tel-omerase [3,4]. The importance of adequate telomerase activity and maintenance of telomere length for replicative potential and aging was initially inferred from studies in primary human fibroblast [5,6]. In culture assays, division of fibroblasts resulted in progressive telomere attrition, culminating in a state of proliferative arrest or cellular senescence after a finite number of cell divisions, a phenomenon known as the Hayflick limit [7]. Excessive telomere shortening prior to the expression of telomerase can lead to chromosome fusion, which has been proposed as a mechanism for chromosome instability [8]. Maser et al. reported the contrasting contributions of telomeres in the initiation and suppression of cancer and reviewed the evidence that radical chromosomal aberrations typify cancer genomes [9].

On the other hand, the stimulation of expression of TERT (the catalytic subunit of telomerase) in cultured human fibroblast stabilized telomere length and endowed the cells with unlimited replicative potential without creating malignant properties [10]. Thus, cellular aging triggered by critical telomere shortening can be prevented or delayed by telomerase reactivation [11]. Induction of telomerase activity that allows indefinite cell proliferation has been documented in different human cells [12]. These crucial *in vitro* studies and others using telomerase knockout mice have been used to investigate telomere dynamics in the processes of aging and in several degenerative diseases in humans. Telomere shortening depends on cell division [13]. Therefore, telomere length not only provides information as an indicator of the replicative history of cells but may also suggest the replicative potential remaining in each cell [14].

Mondello et al. analyzed the length of the terminal restriction fragments (TRF) in fibroblast and blood cells from four healthy subjects >100 years old as well as 11 individuals of different ages. No correlation between mean TRF length and donor age was found. However, as expected, telomere shortening was detected during *in vitro* propagation of fibroblasts from aged subjects, suggesting that telomeres can be far from reaching a critical length [15].

Allsopp et al. examined the rate of telomere shortening in quiescent cells *in vivo* and measured TRF length in brain tissue from adult donors 32–75 years of age. No significant association was observed between TRF length and donor age (*p* = 0.087) in contrast to telomere length shortening that occurs during *in vivo* aging of mitotically active cells (*p* = 0.0001). These observations show that telomere shortening is largely, if not entirely, dependent on cell division and support the end replication problem as a mechanism of this process. Therefore, telomere length can be used as a biomarker for replicative capacity [16].

The purpose of our study was to evaluate telomere length and proliferative potential of peripheral blood mononuclear cells (PBMCs) of young adults compared with older adults. We compared PBMC proliferation before and after stimulation with ConA.

## Methods

Blood samples were obtained from 20 healthy males (20–25 years old) (group Y/young), and 20 males (60–65 years old) (group O/older). All persons included in this study were nonsmokers with no history of alcohol abuse or drug consumption. This protocol was approved by the local Bioethics Committee of the Unidad Médica de Alta Especialidad (UMAE) No. 1 Bajío, Instituto Mexicano del Seguro Social (IMSS), León, Guanajuato, México. Written informed consent was obtained from each volunteer.

### PBMC isolation and culture

PBMCs were isolated by Ficoll-Hypaque density gradient centrifugation (Sigma-Aldrich, St. Louis, MO). PBMCs were labeled with cell tracker dye CFSE (0.5 μM; Molecular Probes, Eugene, OR) to monitor proliferation. Briefly, PBMCs were suspended in PBS at a concentration of 1 × 10^6^/ml, and an equal volume of 1 μM CFSE in PBS was added. PBMCs were incubated in the dark at room temperature for 10 min, centrifuged, and the supernatant discarded. Cells were resuspended in 5 ml of RPMI media and incubated for 30 min at 37°C with 5% CO_2_. CFSE-labeled PBMCs were then cultured with or without 2.5 μg/mL of concanavalin A (ConA, Sigma Aldrich) for 72 h at 37°C, 100% humidity and 5% CO_2_. After that, the percentage of divided cells was determined by flow cytometry analysis with a FACSCalibur™ flow cytometer (Becton-Dickinson, San Jose, CA) by using the Cell Quest software (Becton Dickinson).

### Telomeric measurement

DNA was isolated from PBMCs before and after culture with ConA through phenol–chloroform technique for telomeric measurement. Telomeric length was measured as previously described [17] by PCR amplification with oligonucleotide primers designed to hybridize to the TTAGGG and CCCTAA repeats. The final concentrations of reagents in the PCR were 0.2 SYBR Green I (Molecular Probes), 15 mM Tris–HCl, pH 8.0, 50 mM KCl, 2 mM MgCl_2_, 0.2 mM each dNTP, 5 mM DTT, 1% DMSO and 1.25 U AmpliTaq Gold DNA polymerase. The final telomere primer concentrations were as follows: tel 1, 270 nM; tel 2, 900 nM. The final *36B4* (single copy gene) primer concentrations were 36B4u, 300 nM; 36B4d, 500 nM. Primer sequences (5′→3′) were as follows: tel 1, GGTTTTTGAGGGTGAGGGTGAGGGTGAGGGTGAGGGT; tel 2,TCCCGACTATCCCTATCCCTATCCCTATCCCTATCCCTA; 36B4u, CAGCAAGTGGGAAGGTGTAATCC; 36B4d,CCCATTCTATCATCAACGGGTACAA. [15]. All PCRs were performed using LightCycler® (model 1.5) by Roche thermocycler. The thermal cycling profile for both amplicons began with 95°C incubation for 3 min to activate the AmpliTaq Gold DNA polymerase. For telomere PCR, 40 cycles of 95°C for 15 s, 54°C for 2 min, and for *36B4* PCR, 40 cycles of 95°C for 15 s, 58°C for 1 min were set. LightCycler® (model 1.5) by Roche thermocycler was then used to generate the standard curve for each run and to determine the dilution factors of standards corresponding to the T and S amounts in each sample.

### Statistical analysis

To determine between-group differences, we used Mann–Whitney U test. All data are presented as mean ± SE; *p* < 0.05 was accepted for statistical significance.

## Results

### Telomeric length in PBMCs before and after stimulation with ConA

We measured telomeric length in PBMCs before and after *in vitro* stimulation by RT-PCR technique. Unstimulated cells from group O had shorter telomeres than cells from group Y (*p* = 0.03). Consequently, cells from older adults (group O) did not show any changes of telomeric DNA (*p* = 0.17) and higher levels of senescent (≤6.3 kb) telomeric DNA (*p* = 0.02) (Table 1).

**Table 1:** Telomeric length before and after stimulation in both groups

	**Mean telomere length (kb)**		
	**Before stimulation**	**After stimulation**	***p***
Group Y	12.416 ± 7.67	13.034 ± 20.31	*p* = 0.04
Group O	6.382 ± 6.39	6.295 ± 8.54	*p* = 0.17
	*p* = 0.03	*p* = 0.02	

Note: Significant difference was shown between groups (calculated with Mann–Whitney U test).

Group Y, young adults; group O, older adults.

Data are presented as mean ± SE; *p* <0.05 was considered significant.

Surprisingly, we found increased telomere length in cells from Group Y after *in vitro* stimulation with ConA. Whereas no changes in telomeric length were observed in cells from group O with ConA stimulation (Table 1), cells from group Y are capable of increasing telomere length and, therefore, decreasing the percentage of critically short telomeres.

In both groups (young men and older men), the percentages of CD3+ as well as CD3- cells were similar. However, in group O the CD4+ subset was significantly higher than in group Y, whereas CD8+ population was slightly higher in group Y (data not shown).

### PBMC proliferation and replicative potential

**Figure 1:**
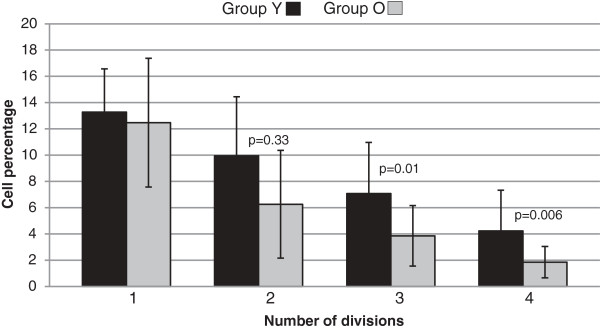
**CFSE proliferation histograms (group Y and group O).** Lymphocyte proliferation with concanavalin A stimulation was significantly different between groups. Number of reached divisions: 1) without divisions, 2) one division, 3) two divisions, and 4) three divisions. Significant difference is shown between groups* (calculated with Mann–Whitney U test). All data are presented as mean ± SE; *p*<0.05 was considered significant.

The capacity of PBMC proliferation after *in vitro* stimulation with ConA was significantly different between groups Y and O. In most cases, more cells from group Y reached more divisions than those in group O (Table 1). Therefore, CFSE-labeled cells from group O undergo fewer cell divisions (Figure 1) and had shorter telomeres than group Y (*p*=0.04) after 72 h of *in vitro* stimulation (Table 1). Coincidentally, cells with a low proliferative response to ConA stimulation (group O) were those with shorter telomeres.

## Discussion

Our findings clearly point to the difference in telomere length and replicative response after ConA stimulation between PBMCs of young subjects and older subjects.

Shortening of telomeres is the cause of replicative senescence of mammalian cells in culture and may be a cause of cellular aging *in vivo*[18]. It has been shown that in some tissues cells suffer telomere shortening during aging in humans [4,19-21].

It is important to note that telomere shortening in aging subjects has been reported in human peripheral blood leukocytes [22,23], in PBMCs [24], and in T cells [25,26]. The significance of this phenomenon is unclear, but it has been suggested that it is related to the diminished immunity that occurs in older age.

Yang et al. investigated the relationship between telomere biology and replicative senescence by measuring replicative capacity and telomere length as a function of donor age in cells from adrenal tissue from donors of different ages. They found an age-related decline in total replicative capacity. These authors confirm the relationship between telomere length, telomerase, and replicative capacity in culture [27]. However, Allsop et al. did not find any relation between TRF length and donor age [5].

Blackburn concluded that telomere length does not act as a mitotic clock and that the presence or absence of telomerase is crucial in maintaining cellular reproductive capacity. The author developed a dynamic two-state model of telomeres in which there was a switch between capped and uncapped states [28]. Enzymatically active telomerase apparently has a protective effect on very short telomeres that, in its absence, would have caused a cessation in cell division [28,29]. Greider [30], a co-discoverer with Blackburn of telomerase, concluded that there is little or no evidence that the changes that cells in culture undergo are the same as those that normal cells undergo with age *in vivo*[31].

This concept assumed an iconic character with the report that ectopic expression of telomerase by a vector greatly extended the lifespan of human cells. That something similar might occur *in vivo* seemed consistent with initial reports that most human somatic tissues lack telomerase activity [31].

Loss of genome integrity and associated DNA damage signaling and cellular checkpoint responses are well-established intrinsic instigators that drive tissue degeneration during aging [32]. Mounting evidence in humans has also provided a strong association of limiting telomeres with increased risk of age-associated disease [33] and with onset of tissue atrophy and organ system failure in degenerative diseases. Controversially, Bestilny et al. reported an inverse relationship between telomere length and progression of immunosuppression, with HIV infection resulting in a 5-fold or greater acceleration of aging of the circulating PBMC component of the immune system [34]. Honda et al. also found that T cells showed an accelerated loss of telomeric DNA in patients with systemic lupus erythematosus [35].

We report here that unstimulated PBMCs from group O have shorter telomeres than group Y. Surprisingly, a 5-kb increase in telomeric length was found in PBMCs from group Y after *in vitro* stimulation with ConA. A previous study showed that restoration of telomerase activity prevented the wide range of degenerative pathologies [36].

Son et al. [37] observed that the capacity for induced telomerase expression in T and B cells is diminished with age. The replicative capacity in young T cells is considerably higher. This is influenced by the size of the telomere because we were able to compare between groups. The relationship between telomere length and replicative capacity before and after ConA stimulation is significantly different between groups. In most cases, more PBMCs reached more divisions in group Y than in group O. Jaskelioff et al. recently demonstrated that multiple aging phenotypes in a mouse model of accelerated telomere loss can be reversed within 4 weeks of reactivating telomerase. The authors speculate that some tissue stem/progenitor cells are retained in a quiescent and intact state, yet they can be enlisted to resume normal repopulating function upon elimination of genotoxic stress of telomeres [12]. Cumulative evidence implicating telomere damage as a driver of age-associated organ decline and disease risk [10,38] and the dramatic reversal of systemic degenerative phenotypes in adult mice observed here support the development of regenerative strategies designed to restore telomere integrity.

## Conclusions

Our findings demonstrate that elongation of telomeres is associated with a higher replicative capacity after stimulation with ConA in young adults. We can only speculate that this phenomenon may have been due to activation of telomerase through series of signaling pathways triggered by ConA. We are not aware that the mechanism of telomere elongation as a consequence of replicative stimulation has yet been studied. However, it may be a promising line of research. It is clear that in cells from older subjects this yet unknown mechanism is impaired.

## Competing interests

The authors declare that there are no conflicts of interest in the elaboration of this investigation.

## Authors’ contributions

BM conceived the study, carried out the molecular genetic studies and participated in its design and coordination and helped to draft the manuscript. FA carried out the molecular genetic studies. SL carried out the flow cytometer and helped to draft the manuscript. SM carried out the molecular genetic studies. LB participated in the design and coordination and helped to draft the manuscript. DA participated in the sequence alignment. All authors read and approved the final manuscript.

## Pre-publication history

The pre-publication history for this paper can be accessed here:

http://www.biomedcentral.com/1471-2318/13/99/prepub
